# National Dementia Plans of Group of Seven Countries and South Korea Based on International Recommendations

**DOI:** 10.1001/jamanetworkopen.2022.40027

**Published:** 2022-11-03

**Authors:** Su Jeong Seong, Bin Na Kim, Ki Woong Kim

**Affiliations:** 1 Department of Psychiatry, Kangdong Sacred Heart Hospital, Seoul, South Korea; 2 Department of International Development Cooperation, Graduate School of Pan-Pacific International Studies, Kyung Hee University, Yongin-si, Gyeonggi-do, South Korea; 3 Department of Neuropsychiatry, Seoul National University Bundang Hospital, Seongnam-si, Gyeonggi-do, South Korea; 4 Department of Neuropsychiatry, Seoul National University, College of Medicine, Seoul, South Korea; 5 Department of Brain and Cognitive Science, Seoul National University College of Natural Sciences, Seoul, South Korea

## Abstract

This quality improvement study investigates whether national dementia plans in Group of Seven (G7) countries and South Korea conform to the recommendations of the World Health Organization and the Organization for Economic Cooperation and Development.

## Introduction

The Organisation for Economic Co-operation and Development (OECD) and the World Health Organization (WHO) proposed 10 key objectives and 7 action areas as guidelines for developing dementia policies.^1,2^ However, to our knowledge, few studies have examined whether existing national dementia plans (NDPs) conform to these international recommendations. This study investigated how well the WHO and OECD recommendations are implemented in existing NDPs in Group of Seven (G7; an intergovernmental political forum) countries and South Korea.

## Methods

We analyzed the latest NDPs of G7 countries (Canada, Germany, Italy, the UK, the US, France, and Japan) and South Korea (Table 1). National dementia plans are defined as standalone comprehensive national strategies, policies, plans, or frameworks to address dementia.^1^ Plans complementary to the most recent NDPs were also analyzed, such as the UK’s “Prime Minister’s Challenge on Dementia 2020: Implementation Plan.” The French “Neurodegenerative Diseases Plan 2014-2019” was identified as the latest NDP because the French “Neurodegenerative Diseases Roadmap 2021-2022” was a transitional extension rather than a new NDP. Because this study did not involve human participants or animals, the Institutional Review Board of Seoul National University Bundang Hospital determined that ethical approval was not required. This study adhered to the SQUIRE reporting guideline.

**Table 1.** National Dementia Plans of Group of Seven Countries and South Korea

Country and title of plan^a^	Period	Release	Responsible authoritative body
Canada			
A Dementia Strategy for Canada^b^	2019^c^	2019	Public Health Agency
Germany			
National Dementia Strategy^b^	2020^c^	2020	Federal Ministry for Family Affairs, Senior Citizens, Women and Youth and the Federal Ministry of Health
Italy			
Italian National Dementia Plan 2015^b^	2015-2018	2014	Ministry of Health
United Kingdom			Department of Health and Social Care
Living Well with Dementia	2009^c^	2009	
Prime Minister’s Challenge on Dementia 2015	2012-2015	2012	
Prime Minister’s Challenge on Dementia 2020^b^; Prime Minister’s Challenge on Dementia 2020 Implementation Plan^b^	2015-2020	2015	
France			
National Plan for “Alzheimer and Related Diseases” 2001-2004	2001-2004	2001	Ministry of Social Affairs, Health and Women’s Rights
National Plan for “Alzheimer and Related Diseases” 2005-2007	2005-2007	2004	
National Plan for “Alzheimer and Related Diseases” 2008-2012	2008-2012	2008	
French Neurodegenerative Diseases Plan 2014-2019^b^	2014-2019	2014	
Neurodegenerative Diseases Roadmap 2021-2022^d^	2021-2022	2021	
United States			
National Plan to Address Alzheimer Disease^b^	2012^e^	2021^f^	Department of Health and Human Services
Japan			
5-Year Plan for Promotion of Dementia Measures (Orange Plan)	2013-2017	2012	Ministry of Health, Labour and Welfare
Comprehensive Strategy to Accelerate Dementia Measures (New Orange Plan)^b^	2015-2020	2015	
South Korea			
Dementia Comprehensive Management Measures	2008-2012	2008	Ministry of Health and Welfare
The 2nd National Dementia Plan 2013-2015	2013-2015	2012	
The 3rd National Dementia Plan 2016-2020	2016-2020	2015	
National Dementia Initiative	2017^c^	2017	
The 4th National Dementia Plan (2021-2025)^b^	2021-2025	2020	

^a^ As of August 2022, the latest national dementia plans were selected for analysis.

^b^ Identified as the latest national dementia plan.

^c^ End date is not specified.

^d^ Because the Neurodegenerative Diseases Roadmap 2021-2022 was a transitional extension to maintain essential action rather than a new national dementia plan, the French Neurodegenerative Diseases Plan has been identified as the latest one.

^e^ Annually updated.

^f^ The latest 2021 update was selected for analysis.

The WHO’s 7 action areas from the “Global Action Plan on the Public Health Response to Dementia”^1^ and the OECD’s 10 key objectives from “Addressing Dementia: The OECD Response”^2^ were integrated into 11 policy targets (prevention, diagnosis, awareness, caregiver support, environment, long-term care, health facilities, end-of-life care, care coordination, research and technology, and information systems) as the comparison framework (eFigure in the Supplement). The first WHO action area (ie, dementia as a public health priority) was not included in the policy targets, because the presence of an NDP may indicate a high public health priority. Next, the NDPs were compared across the 11 targets and policy approaches for each target, which were derived from the WHO action plan and OECD report (eMethods in the Supplement).

## Results

France was the first country and Germany was the most recent country to adopt an NDP. France, the UK, Japan, and South Korea have revised their NDPs several times, while the US has updated its NDP annually since 2012. The 8 countries’ NDPs largely followed the WHO and OECD recommendations and shared common policy themes (Table 2). All the countries covered targets 1 (prevention), 2 (diagnosis), 3 (awareness), 6 (long-term care), 9 (care coordination), 10 (research and technology), and 11 (information systems). However, several policy targets received little attention. Target 8 (end-of-life care) was not addressed in Italy, Canada, or South Korea. Target 4 (caregiver support) was not addressed in Italy and target 7 (health facilities) was not addressed Canada. Target 5 (the appropriate environment) was not addressed in either Italy or Canada.

**Table 2.** Comparing 8 Countries’ National Dementia Plans by Possible Policy Approaches for the 11 Targets^a^

Possible policy approach	Canada	Italy	Germany	UK	US	Japan	France	South Korea
Target 1: dementia risk reduction								
Intensifying preventive awareness	+	+	+	+	+	+	−	+
Intensifying preventive activities (lifestyles, program)	+	−	+	+	+	+	−	+
Research for prevention	+	−	+	+	+	+	+	+
Target 2: early diagnosis of dementia								
Increase the availability and accessibility of diagnostic services	+	+	+	+	+	+	+	+
Provide training to primary care staff in identifying dementia	+	−	+	+	+	+	+	+
Postdiagnostic support to link people to appropriate services	−	−	+	+	−	+	+	+
Target 3: dementia awareness and friendliness								
Public awareness campaigns to reduce stigma	+	+	+	+	+	+	+	+
Targeted education of those who come into contact with PWD	+	−	+	+	+	−	−	+
Dementia education in schools	−	−	−	+	−	+	−	−
Target 4: support for caregivers of PWD								
Increase the availability and uptake of respite care services	+	−	+	+	+	+	+	+
Provide training to caregivers (informal caregivers)	+	−	+	+	+	+	+	+
Provide support to caregivers (focused on peer-to-peer support)	+	−	+	+	−	+	−	+
Target 5: safe and appropriate environment for PWD								
Supporting the improvement of the residential environment	−	−	+	+	−	−	+	+
Introduction of an alternative housing model	−	−	+	+	+	+	+	+
Target 6: safe and high-quality long-term-care services for PWD								
Standardizing long-term-care services	+	−	+	+	+	−	+	+
Training dementia-related workforce for care service	+	+	+	+	+	+	+	+
Monitoring the management of BPSD	−	−	−	+	+	−	−	−
Promoting human rights and decision-making for PWD	+	−	+	+	+	+	+	+
Target 7: adequate health facilities for PWD								
Training staff in recognizing and responding to PWD	−	+	+	+	+	+	−	+
Establishing specialized staff and dedicated wards in hospitals	−	−	+	+	−	−	+	+
Target 8: EOL care for the dignity of PWD								
Improving accessibility to EOL care for PWD	−	−	+	+	−	+	+	−
Training care home staff in EOL care for PWD	−	−	+	+	+	−	+	−
Target 9: coordinated and proactive care closer to home								
Establishing multidisciplinary services	+	+	+	+	+	+	+	+
Providing acute services outside of the hospital	−	−	+	+	+	−	+	−
Target 10: dementia research and innovation								
Promoting user-centered development and assessment of technologies	+	−	+	+	+	+	+	+
Developing measures to facilitate research	+	+	+	+	+	+	+	+
Target 11: information systems for dementia								
Developing national systems to gather information	+	+	+	+	−	−	+	+
Recording and sharing patient data	−	−	−	−	+	+	+	+
Enabling access to data of available services and resources	+	+	+	+	+	+	+	+

Abbreviations: BPSD, behavioral and psychological symptoms of dementia; EOL, end-of-life; PWD, people with dementia.

^a^ A plus sign indicates that the policy approach is addressed in the national dementia plan; a minus sign indicates that the policy approach is lacking in the national dementia plan.

The NDPs shared common policy approaches, such as public campaigns (target 3), making diagnostic services available (target 2), training workforce (target 6), establishing multidisciplinary services (target 9), facilitating research (target 10), and publicizing available services (target 11).

## Discussion

Among several previous comparison studies of NDPs, only 1 used WHO action areas as a framework, which focused on the factors associated with the existence of an NDP.^3^ A systematic comparison of existing NDPs based on international recommendations would provide valuable guidance for the development of NDPs. Combining the WHO’s inclusive action areas and the OECD’s meticulous key objectives, our 11 targets could provide a practical blueprint.

This study had several limitations. First, plans other than NDPs may contain dementia-related policies, especially regarding research, information systems, and long-term care. Second, subnational plans, which were not included in the present study, may complement NDPs in countries with substantial local autonomy.

## Notes

Supplement.
eMethods.
**eFigure.** A Framework for Comparing National Dementia Plans Between Countries
eReferences.
