# Supplementary material for: National Dementia Plans of Group of Seven Countries and South Korea Based on International Recommendations
Source: JAMA Netw Open. 2022 Nov 3;5(11):e2240027. doi: 10.1001/jamanetworkopen.2022.40027 (PMC9634496; doi:10.1001/jamanetworkopen.2022.40027)
Supplement: Supplement. — eMethods. eFigure. A Framework for Comparing National Dementia Plans Between Countries eReferences. [file jamanetwopen-e2240027-s001.pdf]

# Supplementary Online Content

Seong SJ, Kim BN, Kim KW. National dementia plans of Group of Seven countries and South Korea based on international recommendations. *JAMA Netw Open.* 2022;5(11):e2240027. doi:10.1001/jamanetworkopen.2022.40027

**eMethods.**
**eFigure.** A Framework for Comparing National Dementia Plans Between Countries
**eReferences.**

This supplementary material has been provided by the authors to give readers additional information about their work.

© 2022 Seong SJ et al. *JAMA Network Open.*

## eMethods.

### Development of the 11 targets

We integrated WHO's seven action areas from the “Global action plan for dementia[1]” to OECD's ten key objectives from “Addressing dementia: The OECD response[2]” into 11 policy targets (prevention, diagnosis, awareness, caregiver support, environment, long-term care, health facilities, end-of-life care, care coordination, research and technology, information systems) as the framework for analysis (eFigure 1). Very similar policy themes shared by the WHO's action area and OECD's key objectives made this possible.

Each WHO action area was compared to the OECD's ten key objectives. The first WHO action area (i.e., dementia as a public health priority) was not included in the policy targets because the NDP itself indicated a high priority.

The WHO's Area 2 corresponded to the OECD's Objective 3, addressing dementia awareness and a dementia-friendly society. Therefore, Area 2 was selected as Target 3 (awareness and friendliness). Similarly, as WHO's Areas 3 and 5 corresponded to OECD's Objectives 1 and 4, respectively, Areas 3 and 5 were used as Targets 1 (risk reduction) and 4 (caregiver support).

The WHO's Area 4 addressed diagnosis, treatment, care, and support, each of which had been an important policy theme in previous studies. OECD's Objective 2 (early diagnosis), 5 (adequate environment), 6 (long-term care service), 7 (health facilities), 8 (end-of-life care), and 9 (care coordination) could be all included in Area 4. Since Area 4 covers a broad spectrum of policies, we used the OECD's Objectives 2, 5, 6, 7, 8, and 9 as targets 2, 5, 6, 7, 8, and 9, respectively.

Since there is no corresponding OECD policy objective for WHO's Area 6, Area 6 was used as Target 11 (information systems). Although the OECD report mentioned the importance of information systems for dementia, it did not include information systems in the key objectives of dementia policy.

The WHO's Area 7 addressed promoting research and technology development, covering a wider range than the OECD's Objective 10 (technology development). Therefore, Area 7 was used as Target 10 (research and technology).

### Comparative analysis process

A comparative analysis was performed by the authors. First, the contents of each national dementia plan (NDP) were classified by corresponding policy targets. When the NDP included a specific action plan, we evaluated the policy approach to which it corresponded. For example, if an NDP contains an action plan to expand centers for dementia diagnosis, this content belongs to Target 2 (early diagnosis) and is considered policy approach pertaining to "increasing the availability and accessibility of diagnostic services."

Next, another researcher once again classified the content, and if there was a difference in opinion, the final classification was decided through discussion with all, including the corresponding author. If the expressions written in the NDPs were too ambiguous to be classified, an additional search was carried out targeting formal documents, such as previous NDPs or other governmental policy documents. When the NDP did not cover a specific policy target, its contents were inspected again to confirm that there was no relevant information. The corresponding author then checked the final classification results.

© 2022 Seong SJ et al. *JAMA Network Open.*

**eFigure.** A Framework for Comparing National Dementia Plans Between Countries

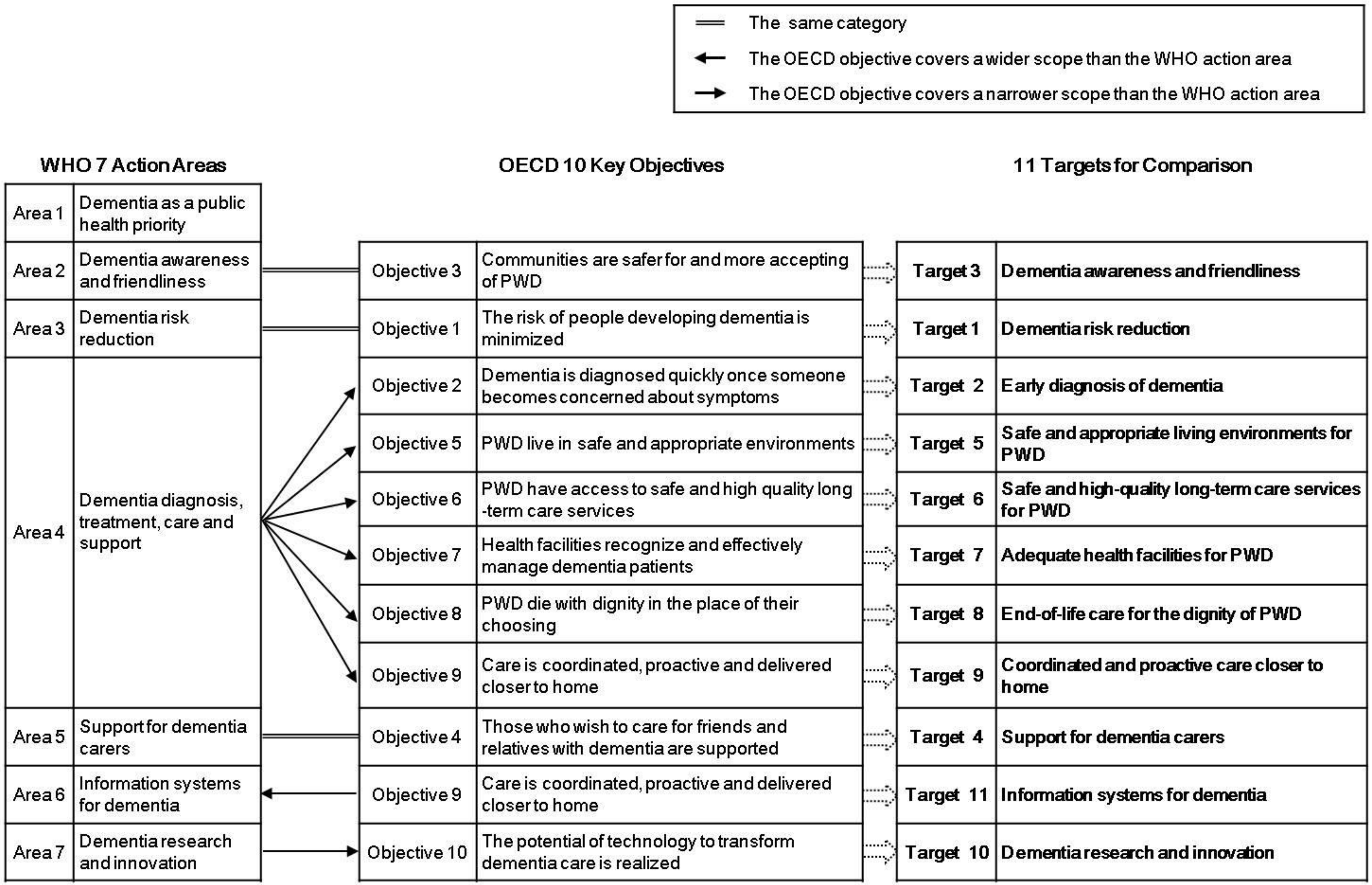


The seven action areas proposed by the WHO and the ten key objectives of the dementia policy proposed by the OECD were mapped and integrated into 11 targets as the framework for comparing the NDPs.

© 2022 Seong SJ et al. *JAMA Network Open.*

## eReferences


1. World Health Organization. Global action plan on the public health response to dementia 2017-2025. https://www.who.int/publications/i/item/global-action-plan-on-the-public-health-response-to-dementia-2017---2025. Accessed Feb 14 2022.

2. OECD Health Policy Studies. Addressing dementia: The OECD response. https://www.oecd.org/els/addressing-dementia-9789264231726-en.htm. Accessed Mar 15, 2021.


© 2022 Seong SJ et al. *JAMA Network Open.*
